# Grain albumin content is affected by 1BL/1RS translocation and alters the processing quality of wheat (*Triticum aestivum* L.)

**DOI:** 10.3389/fpls.2024.1449826

**Published:** 2024-07-22

**Authors:** Shanshan Zhai, Runqi Zhang, Xinhao Meng, Dan Liu, Wenxi Wang, Jiazheng Yu, Chaojie Xie, Jun Ma, Zhongfu Ni, Qixin Sun, Baoyun Li

**Affiliations:** ^1^ College of Agronomy and Biotechnology, China Agricultural University, Beijing, China; ^2^ Tianjin Key Laboratory of Crop Genetics and Breeding, Institute of Crop Sciences, Tianjin Academy of Agricultural Sciences, Tianjin, China

**Keywords:** grain albumin content, QTL, 1BL/1RS translocation, processing quality, wheat

## Abstract

Grain albumin is highly nutritious and closely related to the processing quality of wheat. However, few studies have explored the grain albumin content (GAC) in wheat. This study aims to uncover quantitative trait loci (QTLs) linked to wheat GAC by analyzing a doubled haploid (DH) population derived from common wheat cultivars ShanNong23 and ZhouMai17. We detected six QTLs controlling GAC on chromosomes 1B, 5A, and 6D, with individual QTL explaining 5.78% to 22.29% of the GAC variation. The effect of *QGac.cau-1B.1* on GAC is attributed to the presence of the 1BL/1RS translocation, indicating that the 1BL/1RS translocation increase of GAC compared with the non-1BL/1RS translocation lines. The higher GAC observed in 1BL/1RS lines could be primarily attributed to the increased accumulation of omega-secalin, omega-gliadin, low molecular weight glutenin subunit and ribosomal protein content. Additionally, we also found that the SDS-sedimentation value of whole wheat flour was decreased by adding albumin solution. These results advance our understanding of the genetic basis of GAC and offer novel perspectives for enhancing wheat quality through genetic enhancements.

## 1 Introduction

Bread wheat (*Triticum aestivum* L.) stands as one of the most extensively cultivated crops globally and represents a primary source of plant-based protein in the human diet, contributing 20% of both caloric and protein content to the global human dietary intake (Tilman et al., 2011). Based on protein solubility, wheat proteins can be categorized into four classes: globulin (soluble in dilute salt), albumin (soluble in water), gliadin (soluble in 70% ethanol) and glutenin (soluble in dilute acid or alkali). Among them, gliadin and glutenin constitute the gluten (Snyder, 1907). Wheat flour, as the principal product derived from wheat grains, contains gluten, a constituent absent in other cereal crops. This unique gluten enables the formulation of dough with specific elasticity and extensibility, which is essential for wheat-based food products, such as bread and cookies (Kuchel et al., 2006; Balakireva and Zamyatnin, 2016). Apart from gliadin and glutenin, albumin and globulin are also important in the production and application of wheat. Among them, albumin predominantly accumulate within the embryo and aleurone layers, with a minor fraction found in the endosperm, constituting approximately 9% of the total grain protein content. The albumin, which is primarily consist of structural substances or functionally rich enzymes and proteinase inhibitor factors involved in metabolic processes (Merlino et al., 2009, Merlino et al., 2012), has higher contents of lysine, tryptophan and methionine, with lysine serving as the primary limiting amino acid in the human body. In addition, the content of other amino acids is relatively uniform, contributing to their favorable nutritional value (Wieser et al., 1981). A positive correlation exists between the grain albumin content (GAC) and the total protein content (Yang et al., 1999), whereas an inverse relationship is observed between GAC and processing quality traits, such as SDS-sedimentation value (SSV), dough extensibility (Yang et al., 1999). SSV is widely evaluated to predict the end−use quality of wheat due to its simple measurement method and high correlation with wheat processing quality. Wheat grain albumin plays a crucial role in both the nutritional and processing quality of wheat, however, the current research in this field is relatively limited, and warranted further exploration.

The GAC of wheat is controlled by multiple genes, known as quantitative trait loci (QTLs). In the early years, QTLs associated with GAC have been identified on almost all chromosomes of bread wheat. For instance, a systematic analysis of grain albumin indicated the location of polypeptides of M_r_ 64k, M_r_ 62k and M _r_ 16k on chromosome arms 4DL, 4BS and 3DS respectively (Singh and Skerritt, 2001). Furthermore, an analysis of aneuploid stocks of Chinese Spring wheat has showed that some albumin bands separated by two-step SDS-PAGE were controlled by genes on chromosome arms 4DL, 4AL, 5AL and 5DL (Gupta et al., 1991). QTLs for GAC also have been identified by genome-wide QTL mapping in different populations (Shi et al., 2008; Fang et al., 2018; Zhou et al., 2018). For example, a doubled haploid (DH) population was used to detect albumin-related QTLs on chromosomes 1B, 2A, 2B, 3A, 6A, 6B, 7B and 7D, with a contribution rate of 6.44% to 21.67% (Zhou et al., 2018). In addition, an incremental effect QTL *QAlb2D-3* regulating albumin content was identified using recombinant inbreed lines, contributing 5.03% of albumin content variation (Fang et al., 2018). Despite some previous studies addressing the localization of GAC, the QTL fine mapping for GAC is lacking. Consequently, it is necessary to further specify the locations of the GAC candidate genes using fine-mapping and map-based cloning approaches for novel QTLs.

The short arm of the 1B chromosome (1BS) in wheat is substituted by the chromosome 1RS of rye, resulting the 1BL/1RS translocation lines. 1RS harbors a plethora of disease resistance genes, including leaf rust resistance gene *Lr26* (Friebe et al., 1996), yellow rust resistance genes *Yr9* (Mago et al., 2002) and *YrR212* (Luo et al., 2008), as well as powdery mildew resistance genes *Pm8* and *Pm17* (Mohler et al., 2001). Apart from its commendable disease resistance attributes, the 1BL/1RS translocation lines demonstrate favorable fertility and adaptability (Villareal et al., 1998; Kim et al., 2004; Zhou et al., 2007), and has successfully breed modern varieties such as Veery, Luomai 24, Zhoumai 17, etc., rendering it extensively utilized globally. Nevertheless, investigations indicated a general deficiency in processing quality of 1BL/1RS translocation lines, characterized by diminished SSV (Würschum et al., 2016; Chang et al., 2022), weakened gluten strength (Wieser et al., 2000) and increased dough stickiness (Schwarzlaff et al., 2001), which is not applicable to the bread and other wheat-based food production. The influence of 1BL/1RS translocation lines on wheat quality need further investigation.

In this study, QTLs associated with GAC were mapped using a doubled haploid (DH) mapping population which derived from two wheat cultivars, Shannong23 (SN23) with low GAC and Zhoumai17 (ZM17) with high GAC. Potential key genetic loci were investigated and validated, revealing higher GAC level of 1BL/1RS translocation lines. The results of this study provided a further understanding of the reason for poor processing quality of 1BL/1RS translocation lines.

## 2 Materials and methods

### 2.1 Plant materials and field trials

By using the maize pollination method (De Buyser and Henry, 1980), the DH mapping population consisting of 138 DH lines derived from a cross between wheat cultivars SN23 and ZM17, were constructed and used for QTL mapping of the GAC. All materials above were planted and harvested in four environments: Handan (Hebei province, 36°N, 114°E) in 2019-2020 (E1) and 2020-2021 (E2), Beijing (40°N, 116°E) in 2020-2021 (E3), Qingdao (Shandong province, 36°N, 120°E) in 2020-2021 (E4). In each environment, the materials were planted in a randomized complete block with three replications. Each replication was planted in a 1.0 m long, 0.2 m apart row, with 20 seeds evenly distributed in each row. All management of field trials was consistent with local standard cultivation practice.

To validate *QGac.cau-1B.1*, the F_1_ plants from the cross of SN23 and ZM17 were backcrossed with SN23 and ZM17 separately for three generations with insertion-deletion (InDel) marker-assisted selection (MAS) to generate BC_3_F_1_ population. Subsequently, three key heterozygous lines (Z8 with ZM17 background; S15 and S17 with SN23 background) at QTL-anchored region were identified, and then self-pollinated to BC_3_F_2_. The BC_3_F_2_ were all planted in Handan in 2022-2023 to evaluate the genetic effect of *QGac.cau-1B.1*.

In order to substantiate the impact of 1BL/1RS translocation lines on GAC, two 1BL/1RS translocation lines named as “ER” and “VR” were used. “ER” and “VR” were developed by backcrossing 1BL/1RS translocation lines with the recurrent parent “Pavon76”, a white spring wheat from the International Maize and Wheat Improvement Center (CIMMYT), Mexico. The main differences between “ER” and “VR” lines on the varied sources of the chromosome 1R. The 1RS of “ER” was from the rye variety “E12165” (CIMMYT), while the 1RS of “VR” from “Veery”. The cultivation experiment was carried out in Beijing in 2023. The genotype information of other materials used in this study was introduced in **Supplementary Dataset S3**, which are similar to those described by Liu et al. (2023).

### 2.2 GAC assay and statistical analysis

Grain samples were harvested from the field and stored for approximately 2 months before grinding into whole wheat flour with a Perten LM 3100 experimental mill. Mixed 0.1g of whole wheat flour with 1mL of distilled water, then placed the mixture on a 200 rpm shaker at 37°C for 30 mins, followed by centrifugation at 4000 rpm for 5 mins, and then absorbed the supernatant solution. The above steps were repeated 3 times. Ultimately, mixed the extracted supernatant solution to obtain grain albumin. Subsequently, the grain albumin content was determined by using the Beyotime BCA Protein Assay Kit (Bainor et al., 2011). Three technical repeats were carried out for each sample, ensuring robustness and reliability in the experimental measurements.

Excel was employed to conduct a descriptive statistical analysis. The best linear unbiased predictions (BLUP) values were computed by employing the R package “lme4” (Bates et al., 2018). The genotype treated as a fixed effect, while the environment and replications as random effects. Normal distribution of BLUP value was tested by the Shapiro-Wilk test (α = 0.05) with R software (V.4.3.1; https://www.r-project.org/). The broad-sense heritability ( $H_{B}^{2}$ ) was calculated using the following formula:


$${\text{H}}_{\text{B}}^{2}=\sigma_{\text{g}}^{2}/(\sigma_{\text{g}}^{2}+\frac{\sigma_{\text{ge}}^{2}}{\text{n}}+\frac{\sigma_{\text{er}}^{2}}{\text{nr}})$$

where $\sigma_{g}^{2}$ is variance components for genotype, $\sigma_{ge}^{2}$ denotes the variance of genotype × environment interaction, $\sigma_{er}^{2}$ represents the residual error variance, the variables *n* and *r* correspond to the number of environments and replications, respectively. All the phenotypic differences were analyzed using Student’s *t* test.

### 2.3 Linkage map construction and QTL analysis

Genomic DNA of individual DH lines and corresponding parental lines SN23 and ZM17 was extracted from fresh leaves using the cetyltrimethylammonium bromide (CTAB) method (Allen et al., 2006). Genotyping was conducted using the 55 K wheat single-nucleotide polymorphism (SNP) Genotyping Array (China Golden Marker Co., Beijing, China). The filtering criteria for SNPs for linkage map construction were as follows: Polymorphic SNPs exhibiting variations between the two parents were retained. Subsequently, SNPs with more than 20% missing genotype information, and redundant, co-segregated SNPs with identical genetic distances, were excluded. The resulting SNPs were utilized for the construction of the genetic linkage map. The order and genetic position of markers were calculated based on recombination frequencies using a Regression algorithm and the Kosambi mapping function (Kosambi, 2016). The genetic linkage map was then constructed using JoinMap4.0 and Mapchart v2.32 software (Voorrips, 2002; van Ooijen et al., 2006).

QTL analysis utilized mean trait values obtained from plants grown in individual environments. Windows QTL Cartographer v2.5 software was used for composite interval mapping (CIM) to identify and analyze QTLs (Wang et al., 2010). A threshold LOD value was determined for each of the trials applying the permutation program that was run repeatedly for 1000 times at *P* ≤ 0.05. The threshold value varied with different trials, ranging from 2.3 to 2.5. To be more precise, 2.5 was used as the threshold for all trials. The confidence intervals were established by position ± 2 LOD away from the peak of likelihood ratios.

### 2.4 Genome resequencing and InDel marker development

High-quality genomic DNA from SN23 and ZM17 was sequenced on the Illumina NovaSeq 6000 platform. Subsequently paired-end reads of 2×150 base pairs with an average coverage of 6×for the assembled genome was generated. The alignment of high-quality reads to the Chinese Spring IWGSC RefSeq v1.0 was performed using the Burrows-Wheeler Aligner 0.7.15 program with its default parameters (Li and Durbin, 2009). The InDel markers for QTL analysis were developed based on the sequence difference between SN23 and ZM17 (**Supplementary Dataset S1**). Primer3Plus (https://www.primer3plus.com/) was used for primer design of InDel markers. The PCR reaction system included 5 μl 2×Taq PCR StarMix (GenStar, Beijing, China), 1 μl DNA template (concentration of 50-100 ng), 2 μl InDel primer (mixture of left and right primer, 2 μM), and 2 μl double-distilled water. DNA amplification was programmed for an initial 5 min at 94°C, followed by 35 cycles of 30 s at 94°C, 30 s at 55-58°C, and 30 s at 72°C, and finally 10 min at 72°C. The length of PCR products was analyzed on 8% non-denaturing polyacrylamide gels with silver staining (Marklund et al., 1995).

### 2.5 Transcriptome deep sequencing and IBT quantification proteomics

RNA sequencing (RNA-seq) was performed on 20 days post anthesis (DPA) grains collected from the ER, VR and Pavon76 plants with three independent biological replicates. RNA-seq libraries were generated by BGI Technology Service Co. Ltd. using the DNBSEQ-T7 platform. After screening and trimming, the remaining reads were mapped to the wheat reference genome sequence IWGSC v1.0. Differential expression analysis between ER, VR and Pavon76 was conducted using the Bioconductor package DESeq2 (Anders et al., 2012), criteria of an absolute *P*-value<0.05 and a |log_2_ (fold change)| >1 were employed to identify significantly differentially expressed genes (DEGs).

Grain albumin from ER, VR and Pavon76 was extracted, and then the clear proteins were quantified by using Isobaric tag (IBT) technology (Beijing Genomics institution., Shenzhen, China), with three biological replicates for each sample. Functional annotations of the identified proteins were conducted against the UniProtKB database. The proteins with Fold change >1.5 and *P*-value<0.05 were determined as upregulated proteins. Gene Ontology (GO) annotation was obtained by comparing the identified proteins with the NR (non-redundant) database.

### 2.6 SSV test

SSV was determined according to previously published method (Axford et al., 1979) with some modifications. Weighed 2 g of whole wheat flour into a 35 mL graduated cylinder, added bromophenol blue solution and shaked on a shaker for 5 mins, then added lactic acid-SDS working solution, shaked for 5 mins, removed samples from the shaker and standed for 5 mins. Read sediment volume immediately, which is SSV (mL). All of the above operations were performed at room temperature. In the determination of SSV involving the addition of albumin, the initial step of the measurement substituted the use of distilled water with an albumin solution. Three biological replicates were analyzed for each sample.

### 2.7 Grain protein content test

The grain protein content (GPC) was measured using a near-infrared grain analyzer (Perten DA7200).

## 3 Results

### 3.1 Phenotypic evaluation

**Figure 1:**
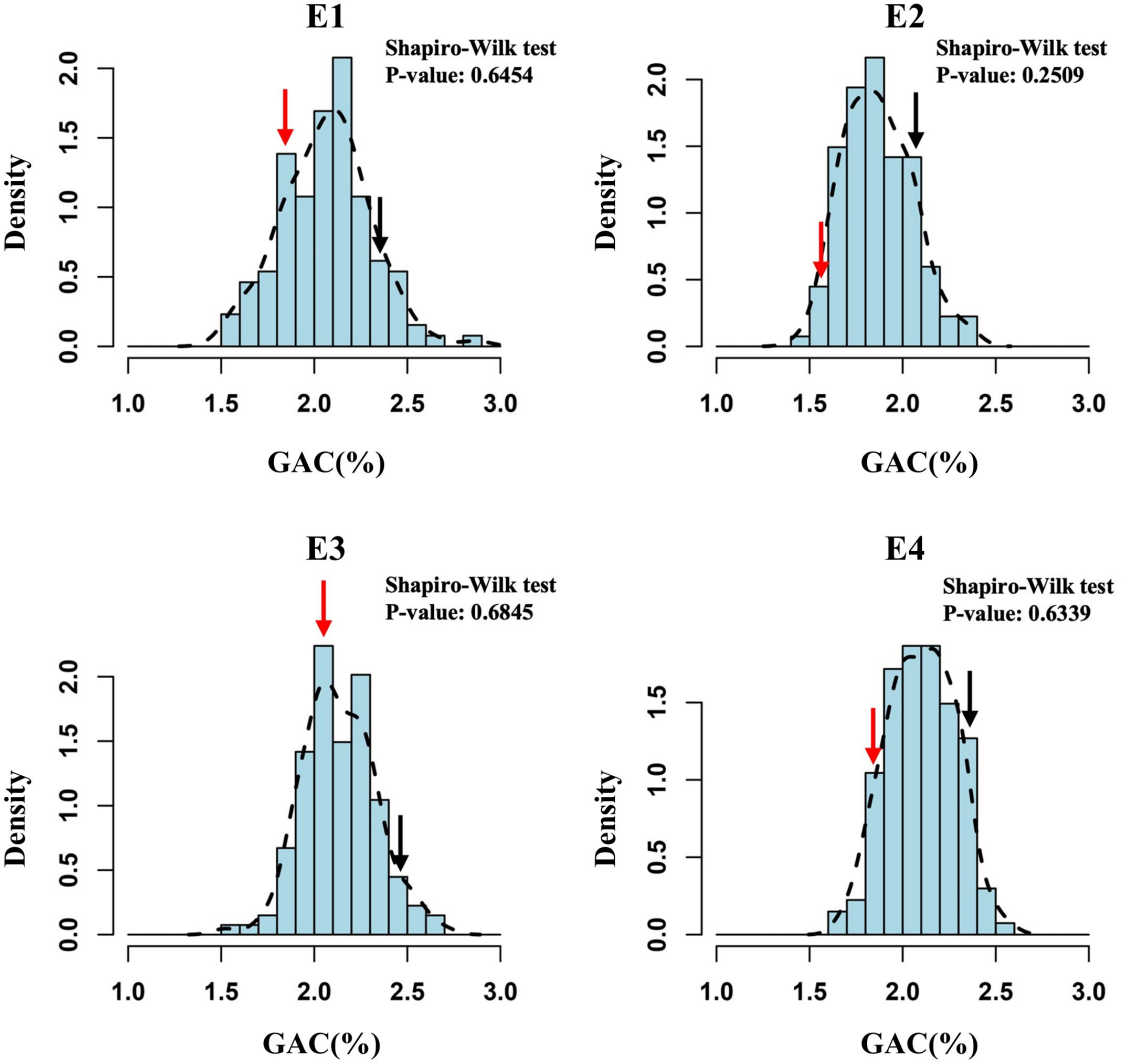
The frequency distribution histograms for grain albumin content (GAC) of the DH population from SN23/ZM17. The *Y*-axis represents the density (the ratio of frequency to group distance) of each trait, and the *X*-axis illustrates the corresponding phenotypic data. Red and black arrow indicate SN23 and ZM17, respectively.

We used the 138 DH lines derived from a cross between wheat cultivars SN23 and ZM17 for QTL mapping of the GAC. The descriptive statistics obtained from multiple environments were listed in **Table 1**. Compared to SN23, ZM17 had significantly higher GAC in all environments. The broad-sense heritability ( $H_{B}^{2}$ ) of GAC was 0.88, indicating that GAC is predominantly controlled by genetic factors (**Table 1**). GAC showed bi-directional transgressive segregation in DH population, suggesting that both SN23 and ZM17 contributed positive alleles for GAC. The result of the Shapiro-Wilk test displayed a normal distribution (*P* > 0.05) in the DH population in four environments, indicating that GAC was regulated by multiple genes (**Figure 1**).

**Table 1:** Statistical analysis of GAC in DH population, SN23 and ZM17 under different environments.

Trait	Environment	SN23	ZM17	DH		$H_{B}^{2}$
		Mean	Mean	Mean ± SD	Range	
GAC (%)	E1	1.89	2.36	2.06 ± 0.23	1.51-2.87	0.88
	E2	1.59	2.08	1.86 ± 0.18	1.44-2.33	
	E3	2.06	2.41	2.13 ± 0.20	1.52-2.70	
	E4	1.88	2.36	2.10 ± 0.18	1.68-2.57	

E1, 2019–2020 Handan; E2, 2020–2021 Handan; E3, 2020–2021 Beijing; E4, 2020–2021 Qingdao. SD, standard deviation.

### 3.2 Genetic linkage map construction

We used the 55 K wheat single-nucleotide polymorphism (SNP) Genotyping Array (China Golden Marker Co., Beijing, China), which containing 33,300 SNPs, for the genotypic analysis of the DH population and corresponding parental lines SN23 and ZM17. A set of 9,380 SNPs was identified between SN23 and ZM17. After excluding polymorphic SNPs with more than 20% missing genotype data and eliminating redundant and co-segregating SNPs with identical genetic distances, a total of 3,339 SNPs were employed for the construction of the genetic linkage map. These SNPs were assigned to 21 linkage groups, spanning all 21 chromosomes of common wheat (**Supplementary Table S1**). The total length of the map was 2,520.87 cM, and the average interval distance between two adjacent markers was 0.75 cM. The extent of the genetic linkage map varied between 26.91 cM for chromosome 5B and 190.41 cM for chromosome 3D. Chromosome 5A exhibited the highest count of distinct loci with 310 markers, while chromosome 7D had the fewest with only 48 markers (**Supplementary Figure S1**, **Supplementary Table S1**).

### 3.3 QTLs detection for GAC

A total of six QTLs associated with the GAC was detected on chromosome 1B, 5A and 6D with LOD > 2.5. QTL that identified in more than two environments were classified as environmentally stable QTLs. Only two environmentally stable QTL were identified on chromosomes 1B and 5A (*QGac.cau-1B.1*, *QGac.cau-5A*), other four QTLs (*QGac.cau-1B.2*, *QGac.cau-1B.3*, *QGac.cau-6D.1*, *QGac.cau-6D.2*) could only be detected in a singular environment (**Table 2**, **Supplementary Figure S2**).

**Table 2:** QTLs for the GAC detected in all environments in the DH population.

QTL	Environment	LOD2_ Interval(cM)	Physical interval (Mb)	LOD	*R^2^* (%)	*Add*
*QGac.cau-1B.1*	E1	0.00-4.10	54.9-487.7	3.11	7.41	0.15
	E2	0.40-3.00	14.7-470.9.	6.59	22.29	0.29
	E3	0.00-2.90	54.9-470.9.	5.02	11.87	0.14
	E4	0.00-2.40	54.9-470.9	6.55	17.10	0.17
	BLUP	0.00-2.20	54.9-470.9	7.05	19.22	0.20
*QGac.cau-1B.2*	E2	11.50-13.80	522.4-541.3	3.29	7.60	-0.17
*QGac.cau-1B.3*	BLUP	5.80-11.70	463.5-530.0	3.51	9.89	0.14
*QGac.cau-5A*	E2	91.80- 99.10	552.2-559.1	2.67	6.54	0.10
	E3	86.60-96.90	537.9-555.3	2.56	5.78	0.10
	E4	87.00-95.90	539.5-555.1	3.49	8.28	0.10
*QGac.cau-6D.1*	E3	39.00-46.30	335.9-418.2	3.65	8.41	0.18
*QGac.cau-6D.2*	E3	56.90-60.20	429.7-436.9	5.48	13.02	-0.22

Physical interval represents the position of the QTL region in the reference genome of ‘Chinese Spring’ (RefSeq v1.0). R2, phenotypic variation explained by the identified QTL. Add, additive effect. Positive and negative values of the Add indicate increasing effects from the ZM17 and SN23 alleles, respectively.

The major QTL *QGac.cau-1B.1* with LOD score of 3.11-7.05 in the four environments and the BLUP data, which was flanked by the markers *AX-111647159* and *AX-111036572*. The positive allele of *QGac.cau-1B.1* was derived from ZM17, and contributed to 7.41%-22.29% of the phenotypic variation for the GAC. The physical interval between flanking markers was 14.7 Mb to 487.7 Mb, which covers the entirety spans of 1BS (**Table 2**; **Supplementary Figure S2**). Another major stable QTL, *QGac.cau-5A*, fixed by markers *AX-108877013* and *AX-109494596* on chromosome 5A with LOD ≥ 2.56, was identified in three environments (E2, E3, and E4). It had positive allele contributed by ZM17, and explained 5.78%-8.28% of the total phenotypic variation of GAC. The physical interval between flanking markers was 537.9 Mb to 559.1 Mb (**Table 2**, **Supplementary Figure S2**).

### 3.4 Validation of the *QGac.cau-1B.1*

**Figure 2:**
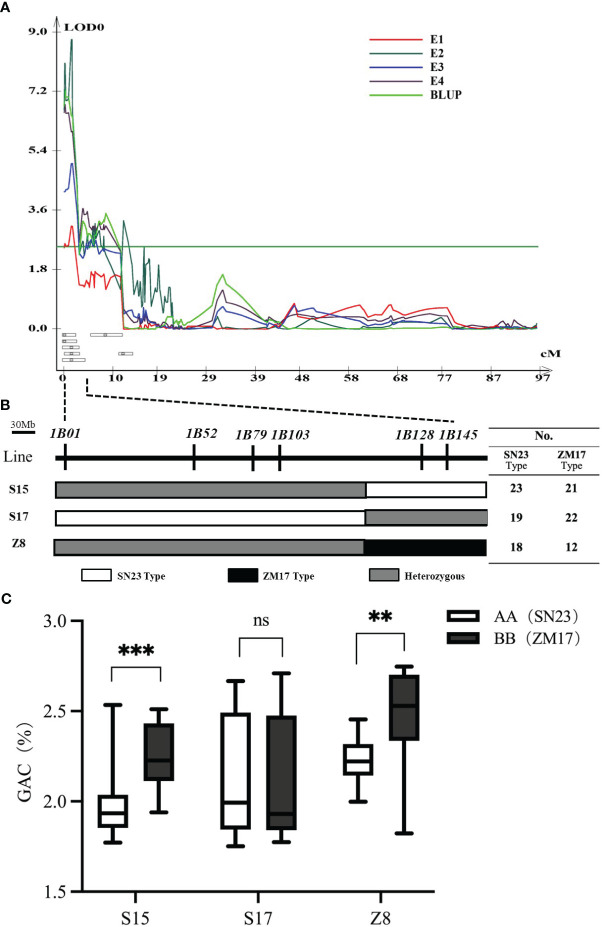
Verification of *QGac.cau-1B.1*. **(A)** QTL mapping for grain albumin content (GAC) in the four environments (E1-E4) and BLUP of chromosome 1B. **(B)** Three individuals with heterozygous intervals covering different genetic segments of the *QGac.cau-1B.1* from the BC_3_F_1_ population. The table on the right lists the number of SN23 type and ZM17 type used for phenotypic examination in the BC_3_F_2_ population derived from each individual. **(C)** The comparisons of grain albumin content between AA (SN23) and BB (ZM17) within each BC_3_F_2_ population. Significant differences are indicated by **P< 0.01; ***P< 0.001 (Student’s *t* test).

To validate the genetic effect of the *QGac.cau-1B.1* (**Figure 2A**), six InDel markers (*1B01*, *1B52*, *1B79*, *1B103*, *1B128* and *1B145*) within this region were developed according to the resequencing results of two parents. These markers were subsequently used to screen three key individuals (S15, S17 and Z8) from the BC_3_F_1_ population with heterozygous intervals covering different genetic segments (**Figure 2B**), and isolated population BC_3_F_2_ with corresponding homozygous intervals covering different genetic segments. The result of Student’s *t* test suggested that the BC_3_F_2_ offsprings of S15 and Z8, which having the same alelle as ZM17 (BB) exhibited significantly higher GAC than those with the SN23 (AA) alelle (**Figure 2C**). However, we detected no significant differences in GAC between ZM17 genotype and SN23 genotype in S17 BC_3_F_2_ population. Taken together, *QGac.cau-1B.1* was located between the molecular markers *1B01* and *1B128*, the physical interval was 1.0 Mb-457.4 Mb according to the IWGSC RefSeq v1.0, which covered the entirety of the 1BS. The accuracy of the *QGac.cau-1B.1* interval was validated.

### 3.5 Comparison of GAC between 1BL/1RS translocation lines and non-1BL/1RS translocation lines

**Figure 3:**
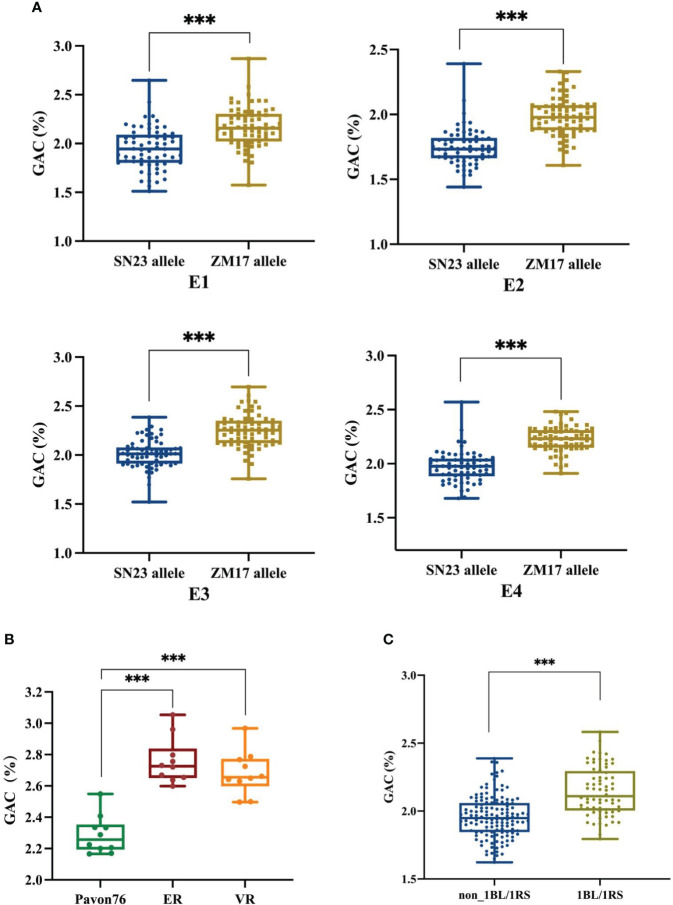
The effect of 1BL/1RS translocation and non 1BL/1RS translocation alleles on grain albumin content (GAC). **(A)** Comparative analysis of grain albumin content between 1BL/1RS translocation lines and non-1BL/1RS translocation lines among 138 DH lines in the four environments. **(B)** Analyzing the grain albumin content in 1BL/1RS translocation materials ER and VR in comparison with their near isogenic line: Pavon76. **(C)** Comparison of grain albumin content within 136 non-1BL/1RS translocation materials versus 68 1BL/1RS translocation materials. Significant differences are indicated by ***P< 0.001 (Student’s *t* test).

According to the resequencing results of ZM17, it was wheat-rye 1BL/1RS chromosome translocation line, and it contributed positive alleles for *QGac.cau-1B.1*. Therefore, we speculated that the observed impact of *QGac.cau-1B.1* on GAC could be attributed to the presence of the 1BL/1RS translocation. We utilized the marker *Bmac0213* linked to *Sec-1* locus in rye (Nagy et al., 2003) to genotype DH population (**Supplementary Figure S2**). The results demonstrated a significant higher average GAC within lines carrying the ZM17 allele compared to those of lines with the SN23 allele across four distinct environments (**Figure 3A**). This result suggested that the impact of *QGac.cau-1B.1* on GAC was associated with the 1BL/1RS translocation. To further substantiate the impact of the 1BL/1RS translocation on GAC, we utilized two 1BL/1RS translocation lines, ER and VR, which was formed a pair of near-isogenic line with Pavon76, respectively. By analyzing the GAC of these lines, we observed a significant higher GAC in both ER and VR compared to Pavon76 (**Figure 3B**). Concurrently, an analysis of the GAC was conducted in a population of 204 wheat germplasms, comprising 136 non-1BL/1RS translocation materials and 68 1BL/1RS translocation materials. The results revealed a significant higher GAC in materials containing the 1BL/1RS translocation compared to those without the 1BL/1RS translocation (**Figure 3C**). The above results suggested that GAC was influenced by the 1BL/1RS translocation, and higher levels of GAC were observed when the translocation is present.

### 3.6 Grain albumin component analysis

**Figure 4:**
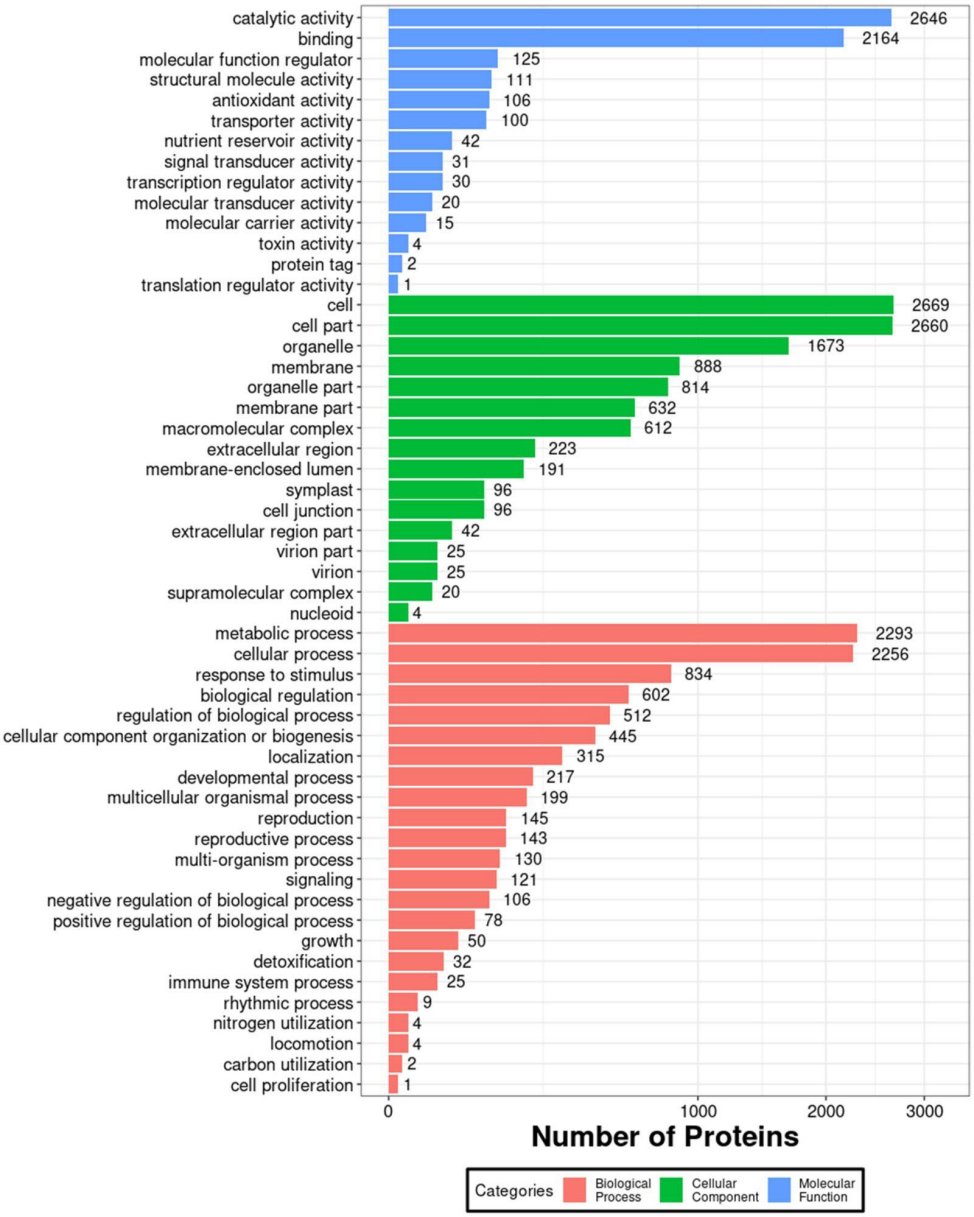
Enriched GO annotation map of all grain albumin identified by the IBT quantification proteomics.

In order to further investigate the impact of 1BL/1RS translocation on grain albumin, a comparative proteomic analysis was conducted between the grain albumin of VR, ER, and Pavon76. A total of 5283 proteins were identified under a 1% FDR filtering criterion. GO annotation analysis of all identified proteins revealed that, in terms of molecular function, grain albumin was predominantly associated with “catalytic activity” and “binding”. In relation to cellular components, the majority of grain albumin was concentrated in “cell” “cell part” and “organelle”. Regarding biological processes, grain albumin was primarily involved in “metabolic process” and “cellular process” (**Figure 4**). These findings underscore the crucial role of grain albumin in various metabolic activities during the developmental stages of wheat, shedding light on its significant contributions in wheat growth and development.

Among the identified proteins, 229 proteins in ER and 183 proteins in VR exhibited significantly higher protein levels compared to Pavon76, and both exhibited the highest number of upregulated proteins on the chromosome 1B (**Supplementary Dataset S4**, **S5**). We specifically focused on the upregulated proteins on 1BS, it was found that the predominant component was omega-secalin. Furthermore, this group encompassed omega-gliadin, low molecular weight glutenin subunit (LMW-GS), ribosomal proteins, and a diverse array of enzymes (**Table 3**). At the same time, we conducted transcriptome analyses of grains collected from the ER, VR and Pavon76 on 20 DPA, when the transcription levels of genes encoding grain protein generally higher. Genes exhibiting differential expression also include those responsible for encoding omega-secalin, omega gliadin, LMW-GS, and ribosomal protein (**Table 4**). This indicated that these proteins have high solubility in water, which may be a primary contribution factor to the higher GAC in 1BL/1RS translocation lines compared to non-1BL/1RS translocation lines.

**Table 3:** Grain albumin upregulated expression in ER and VR on 1BS compared with Pavon76.

ER VS Pavon76		VR VS Pavon76	
Protein_ID	Description	Protein_ID	Description
A0A159KHX4	Omega-secalin	A0A159KHX4	Omega-secalin
C0KEI1	Omega-gliadin (Fragment)	C0KEI1	Omega-gliadin (Fragment)
D3UAL7	Low molecular weight glutenin subunit B3-2 (Fragment)	D3UAL7	Low molecular weight glutenin subunit B3-2 (Fragment)
A0A3B5YUE8	60S ribosomal protein L11	A0A3B5YUE8	60S ribosomal protein L11
A0A3B5YU22	PLAT domain-containing protein	A0A3B5YU22	PLAT domain-containing protein
A0A159KHW5	Omega-secalin	A0A159KIB1	Omega-secalin
A0A3B5YSU3	Glutamate–cysteine ligase	A0A159KI10	Omega-secalin
		A0A159KI88	Omega-secalin
		C1KFY5	Putative omega secalin
		C1KDF4	Putative omega secalin
		A0A3B5YS72	Protein ABIL1
		A0A3B5YQY0	Subtilisin-chymotrypsin inhibitor-2A

Shaded areas represent grain albumin fractions co-upregulated in VR and ER relative to Pavon76.

**Table 4:** Grain albumin proteomics and transcriptome overlapping genes on 1BS of VR and ER.

Gene_ID	ER Protein_ID	VR Protein_ID	Protein Description
*TraesCS1B02G041928*	C0KEI1	C0KEI1	Omega-gliadin (Fragment)
	A0A159KHX4	A0A159KHX4	Omega-secalin
	A0A159KHW5		Omega-secalin
		A0A159KIB1	Omega-secalin
		A0A159KI10	Omega-secalin
		A0A159KI88	Omega-secalin
		C1KFY5	Putative omega secalin
		C1KDF4	Putative omega secalin
*TraesCS1B02G011600*	D3UAL7	D3UAL7	Low molecular weight glutenin subunit B3-2 (Fragment)
*TraesCS1B02G144300*	A0A3B5YUE8	A0A3B5YUE8	60S ribosomal protein L11
*TraesCS1B02G043800*		A0A3B5YQY0	Subtilisin-chymotrypsin inhibitor-2A

### 3.7 The impact of grain albumin on SSV

**Figure 5:**
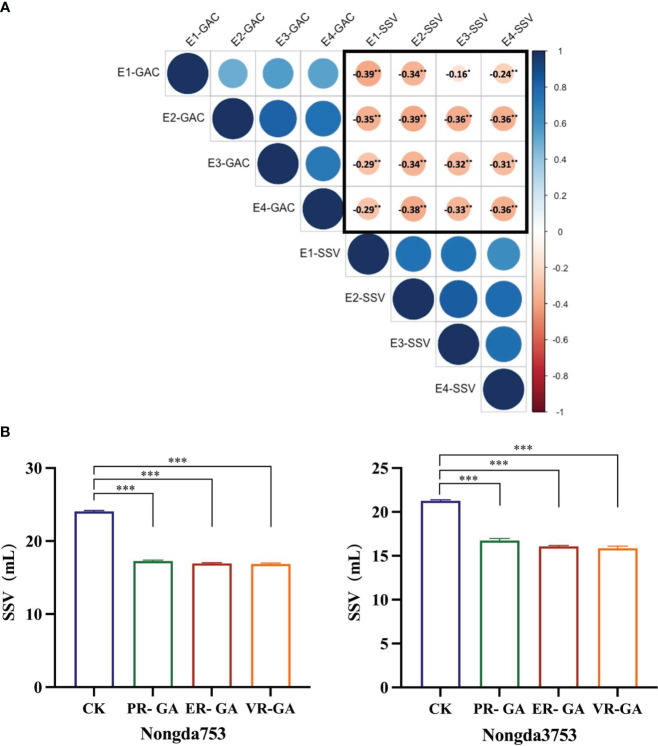
The effect of grain albumin content (GAC) on SDS-sedimentation value (SSV). **(A)** The correlation between grain albumin content and SDS-sedimentation value of DH population under the four environments. The values circled in black represent Pearson’s correlation coefficients. * and ** correlation are significant at *P<* 0.05, *P<* 0.01, respectively (Student’s *t* test). **(B)** The impact of grain albumin on the SDS-sedimentation value of whole wheat flour from Nongda 3753 and Nongda 753. Significant differences are indicated by ***P< 0.001 (Student’s *t* test).

SSV of whole wheat flour serves as a comprehensive quality characteristic that mirrors both the quantity and quality of protein in wheat grains, which is closely related to the gluten strength of wheat and the processing quality of bread and other bakery products (Axford et al., 1979). We measured the whole wheat flour SSV and GPC of 138 DH populations across four environments. Correlation analysis indicated a positive relationship between GAC and GPC, while no significant correlation was found between SSV and GPC (**Supplementary Table S2**). Notably, the analysis consistently revealed a significant negative correlation between SSV and GAC in all environments (**Figure 5A**, **Supplementary Table S2**), suggesting that the increasing GAC is associated with a concurrent reduction in the SSV. In addition, the distilled water used in the first step of the SSV determination procedure was replaced with grain albumin from ER, VR and Pavon76 to study the effect of grain albumin on the SSV of whole wheat flour from strong gluten wheat varieties Nongda 3753 and Nongda 753. It was found that there was a significant decrease in the SSV. Further analysis revealed that the grain albumin from ER and VR caused a more significant decrease in SSV relative to that of Pavon76 (**Figure 5B**). The reason for the above phenomenon may be attributed to the higher GAC in 1BL/1RS translocation lines ER and VR compared to Pavon76. Therefore, the higher GAC in the 1BL/1RS translocation lines, relative to the non-1BL/1RS translocation lines likely contributes to the reduced processing quality.

## 4 Discussion

The albumin in wheat grains is a water-soluble protein with a balanced amino acid composition, thus possessing high nutritional value. Furthermore, it is intricately linked to the quality of wheat. Given the association between albumin and wheat quality, several studies have been conducted (Yang et al., 1999; Merlino et al., 2009; Žilić et al., 2011). However, there remains a notable dearth of reports on this subject. In the present study, we identified 6 QTLs for the GAC on chromosomes 1B, 5A and 6D by using the DH population derived from SN23/ZM17. Among which, *QGac.cau-1B.1* and *QGac.cau-5A* were main environmental stability QTLs (**Table 2**; **Supplementary Figure S2**). Zhou et al. (2018) detected 5 QTLs controlling GAC on chromosome 1B using DH population. These QTLs were distributed on the entire chromosome 1B based on their physical positions. Notably, in our study, *QGac.cau-1B.1* mainly spans the short arm of chromosome 1B, with its enhancing allele originating from ZM17, which is associated with the 1BL/1RS translocation lines. Our genetic studies indicated that 1BL/1RS translocation lines had higher GAC compared with non-1BL/1RS translocation lines, which has been rarely reported in previous studies. Furthermore, Gupta et al. (1991) used aneuploid stocks of Chinese Spring wheat to identify genes controlling albumin on 4DL, 4AL, 5AL and 5DL, and immunoblot analysis revealed that the protein bands controlled by chromosome arms 4DL, 4AL and 5AL were wheat β-amylase of the grain and may be encoded at the β-*Amy*-*1* loci on these arms. However, in our study, the region encompassed within *QGac.cau-5A* does not harbor a gene encoding β-amylase, suggesting it might not correspond to the same locus, which warrants further investigation in subsequent research.

Transferring fragments carrying advantageous genes (such as disease resistance, stress tolerance, high yield) from wild relatives into the wheat background has become a critical approach for broadening the genetic basis, expanding the genetic variation types, and selecting breakthrough new varieties. For example, the Trititrigia Tzvelev translocation lines derived from the cross between octaploid Trititrigia Tzvelev and common wheat is mainly used for disease resistance, particularly in combating powdery mildew and rust (Cui et al., 2021; Yang et al., 2021; Li et al., 2022). The 1RS segment of rye carries many excellent genes, the 1BL/1RS translocation line possesses distinct advantages in disease resistance, stress tolerance, and high yield potential (Villareal et al., 1998; Kim et al., 2004; Zhou et al., 2007; Crespo-Herrera et al., 2017). Although the 1BL/1RS translocation line is widely used in wheat breeding and production, it is commonly associated with subpar processing quality (Barbeau et al., 2001; Graybosch, 2001; Li et al., 2009; Zhao et al., 2012). Previous studies have revealed that the main reason is that rye 1RS carries the *Sec-1* locus encoding secalins, which replaces the *Gli-B1* and *Glu-B3* loci of 1BS, reducing the expression of important gliadin and LMW-GS resulting in quantitative and qualitative changes of gluten components (Martín and Carrillo, 1999; He et al., 2005; Zhang et al., 2007; Howell et al., 2014; Chai et al., 2016). Grain albumin has a negative impact on the processing quality of wheat (Yang et al., 1999). Therefore, based on results from current study, we suggested that high grain albumin content in 1BL/1RS translocation lines may be another reason for their poor processing quality. Accompanying unfavorable genes may be introduced when advantageous genes from wheat wild relatives are transferred into common wheat. Thus, efforts should minimize the adverse effects of non-compensating translocations for breeding of translocation lines in future.

Wheat protein plays a crucial role in determining processing quality. Currently, wheat proteins are commonly categorized into four groups: globulin, albumin, gliadin and glutenin, following the osborne classification system (Snyder, 1907). Achieving a harmonious balance between the quantity and quality of each protein is a pivotal aspect in breeding for enhanced wheat quality. According to the results of the proteomic and transcriptome analyses, the main reason for the increased GAC in the 1BL/1RS translocation lines is the increased content of omega-secalin, omega-gliadin, LMW-GS and ribosomal protein (**Tables 3**, **4**). It is noteworthy that omega-secalin and omega-gliadin are predominantly alcohol-soluble proteins, while LMW-GS is primarily soluble in dilute acid and alkali solutions (Snyder, 1907; Chai et al., 2016). However, all these proteins were detected in the grain albumin fraction in this study (**Table 3**). This indicates that these proteins possess some degree of water solubility, suggesting that the classification of wheat proteins based on solubility characteristics may have limitations. Therefore, there may be a necessity for a more scientifically grounded wheat protein classification system to serve wheat quality breeding.

Numerous traits are utilized to assess the processing quality of wheat, such as SSV, gluten index, dough rheological properties, and dough extensibility. Among these, SSV plays an important role in evaluating wheat processing quality (Axford et al., 1979). It exhibits significant correlations with various quality indicators including protein content and bread volume (Ozturk et al., 2008; Li et al., 2009; Würschum et al., 2016). Moreover, due to its high heritability and small amount of test samples, it is often used by wheat quality breeders as a reliable indicator for selecting early-generation wheat materials (Carter et al., 1999). Previous studies on QTL mapping of SSV has revealed that the presence of the 1BL/1RS translocation significantly impacted SSV, which the SSV tends to be lower in 1BL/1RS translocation lines (Li et al., 2009; Würschum et al., 2016; Chang et al., 2022). In our study, correlation analysis revealed a negative correlation between GAC and SSV (**Figure 5A**). Previous research has indicated a positive correlation between the albumin content and the total protein content of wheat grains (Yang et al., 1999), which is consistent with the findings of this study (**Supplementary Table S2**). But the increase in the albumin content of wheat grains may relatively reduce the content of other vital protein components, such as gliadin and glutenin, which are crucial to the SSV (Sharma et al., 2020), thereby causing a decrease in the SSV. However, this hypothesis requires further empirical validation to confirm and elucidate the underlying mechanisms. Additionally, substituting the distilled water conventionally used in the initial step of SSV determination with a grain albumin solution resulted in a significant decrease in the SSV of whole wheat flour (**Figure 5B**). Notably, this reduction became more pronounced with higher concentrations of the albumin solution. Therefore, selecting materials with lower GAC will be crucial to ensuring high SSV in high-quality wheat breeding programs in future, which in turn will enhance processing quality and enable the cultivation of strong-gluten wheat suited for food products such as bread. Conversely, by selecting higher GAC materials, weak-gluten wheat that are more suitable for food processing such as pastries.

Based on the results of this study, higher albumin content observed in 1BL/1RS translocation lines compared with non-1BL/1RS translocation lines can be primarily attributed to elevated levels of omega-secalin, omega-gliadin, LMW-GS and ribosomal protein. Omega-secalin, being abundant in glutamine, enhances the water absorption of flour, consequently leading to increased stickiness of the dough (Martín and Carrillo, 1999). Moreover, some studies have found that gliadin negatively affects the functionality of gluten, dough, and flour-based products, reducing the expression and content of gliadin, which often resulting in the enhancement of wheat quality traits (Gil-Humanes et al., 2012; Altenbach et al., 2014; Li et al., 2018). Simultaneously, LMW-GS acting as crucial functional counterparts to HMW-GS, play an important role in wheat quality. Variations in their content can induce alterations in the protein component structure, consequently impacting the processing quality of wheat (D’Ovidio and Masci, 2004; Zhang et al., 2012). Ribosomal proteins play an important regulatory role in plant life activities, but whether they have an impact on wheat quality requires further experimental verification (Wang et al., 2020). To sum up, our findings suggested that the reduction of SSV observed in the presence of albumin is mainly own to affected protein composition and disrupted gluten structure, thereby negatively affecting the quality traits of wheat. Nevertheless, further research is necessary to elucidate the precise impact.

## 5 Conclusions

This study identified six QTLs related to GAC in wheat using DH populations derived from common wheat varieties SN23 and ZM17. Proteomic and transcriptome analysis showed that high albumin content in wheat varieties with the 1BL/1RS translocation may be mainly caused by increased levels of omega-secalin, omega-gliadin, LMW-GS and ribosomal protein. It is noteworthy that there is a negative correlation between GAC and SSV, and the albumin solution can significantly reduce the SSV. These results suggested that in future breeding programs, selecting 1BL/1RS translocation lines with reduced albumin content could ensure improved processing quality while ensuring stress resistance and yield potential. Additionally, the addition of albumin had a potential impact on the processing quality of wheat flour, which deserves further exploration and research to fully understand its mechanism, thus offering novel insights for enhancing wheat quality in the future.

## Data availability statement

The RNA-seq data in this study have been deposited into the National Center for Biotechnology Information (NCBI) Sequence Read Archives (SRA) with accession code PRJNA900521.

## Author contributions

SZ: Data curation, Formal Analysis, Writing – original draft. RZ: Data curation, Investigation, Writing – review & editing. XM: Investigation, Writing – review & editing. DL: Investigation, Writing – review & editing. WW: Data curation, Software, Writing – review & editing. JY: Data curation, Software, Writing – review & editing. CX: Conceptualization, Writing – review & editing. JM: Writing – review & editing. ZN: Resources, Supervision, Writing – review & editing. QS: Resources, Supervision, Writing – review & editing. BL: Conceptualization, Project administration, Writing – review & editing.
