# Supplementary material for: Grain albumin content is affected by 1BL/1RS translocation and alters the processing quality of wheat (Triticum aestivum L.)
Source: Front Plant Sci. 2024 Jul 22;15:1449826. doi: 10.3389/fpls.2024.1449826 (PMC11301643; doi:10.3389/fpls.2024.1449826)
Supplement: Supplementary file 1 [file DataSheet_1.docx]

Supplementary Material

# Supplementary Data

**Table S1** Statistics of the constructed genetic map using the doubled haploid (DH) population

| chromosome | No. of linkage groups | No. of markers | No. of loci | Length (cM) | Average locus interval (cM) |
| --- | --- | --- | --- | --- | --- |
| 1A | 1 | 407 | 121 | 43.92 | 0.36 |
| 1B | 1 | 665 | 280 | 96.57 | 0.34 |
| 1D | 1 | 339 | 151 | 90.43 | 0.60 |
| 2A | 1 | 360 | 177 | 94.79 | 0.54 |
| 2B | 1 | 795 | 264 | 155.92 | 0.59 |
| 2D | 1 | 278 | 93 | 83.93 | 0.90 |
| 3A | 1 | 571 | 216 | 173.95 | 0.81 |
| 3B | 1 | 716 | 225 | 184.80 | 0.82 |
| 3D | 1 | 103 | 66 | 190.41 | 2.89 |
| 4A | 1 | 241 | 120 | 130.67 | 1.09 |
| 4B | 1 | 423 | 134 | 118.58 | 0.88 |
| 4D | 1 | 142 | 62 | 115.03 | 1.86 |
| 5A | 1 | 1195 | 310 | 189.35 | 0.61 |
| 5B | 1 | 279 | 84 | 26.91 | 0.32 |
| 5D | 1 | 78 | 63 | 148.07 | 2.35 |
| 6A | 1 | 258 | 79 | 83.51 | 1.06 |
| 6B | 1 | 786 | 252 | 130.78 | 0.52 |
| 6D | 1 | 224 | 148 | 121.78 | 0.82 |
| 7A | 1 | 461 | 210 | 139.83 | 0.67 |
| 7B | 1 | 875 | 236 | 148.84 | 0.63 |
| 7D | 1 | 184 | 48 | 52.80 | 1.10 |
| A | 7 | 3086 | 1112 | 812.10 | 0.73 |
| B | 7 | 4539 | 1475 | 862.40 | 0.58 |
| D | 7 | 1348 | 631 | 802.45 | 1.27 |
| Total | 21 | 9380 | 3339 | 2520.87 | 0.75 |

**Table S2** Pearson’s correlation analysis among GAC, GPC and SSV in BLUP environment.

| Traits | GAC | GPC | SSV |
| --- | --- | --- | --- |
| GAC | 1 |  |  |
| GPC | 0.27* | 1 |  |
| SSV | -0.38** | 0.07 | 1 |

**Correlation is significant at the 0.01 level ，*Correlation is significant at the 0.05 level

**
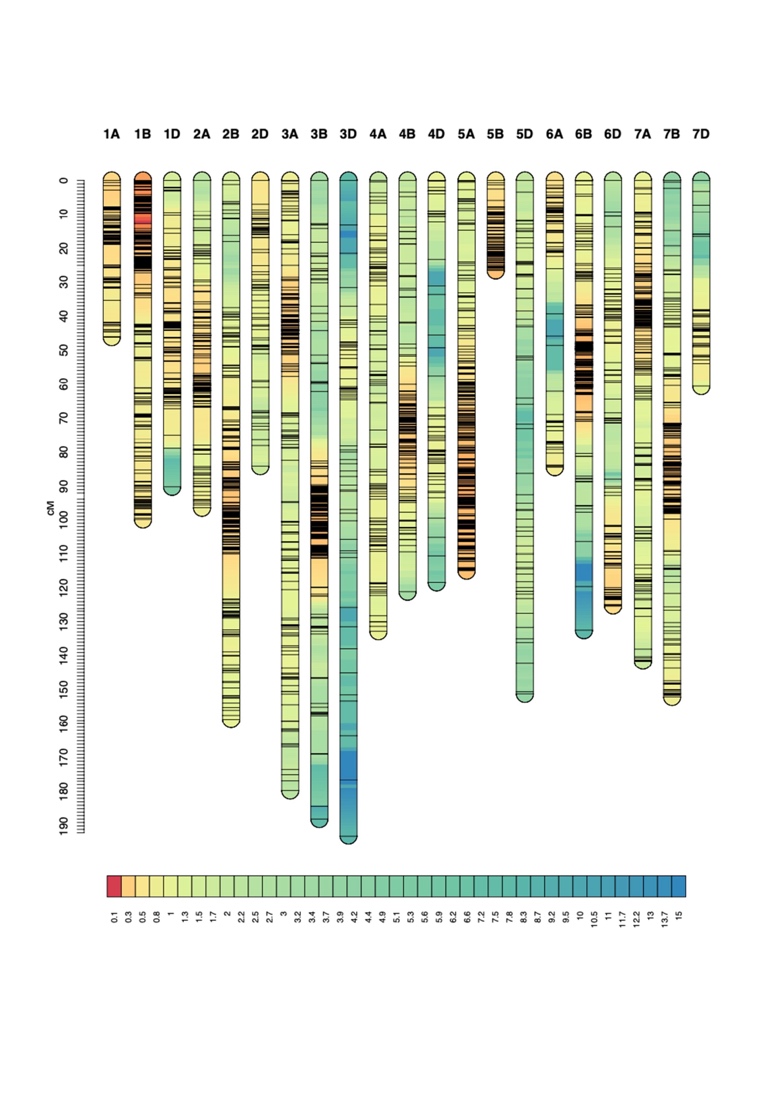
**

**Fig. S1** High-density genetic map of doubled haploid (DH) population


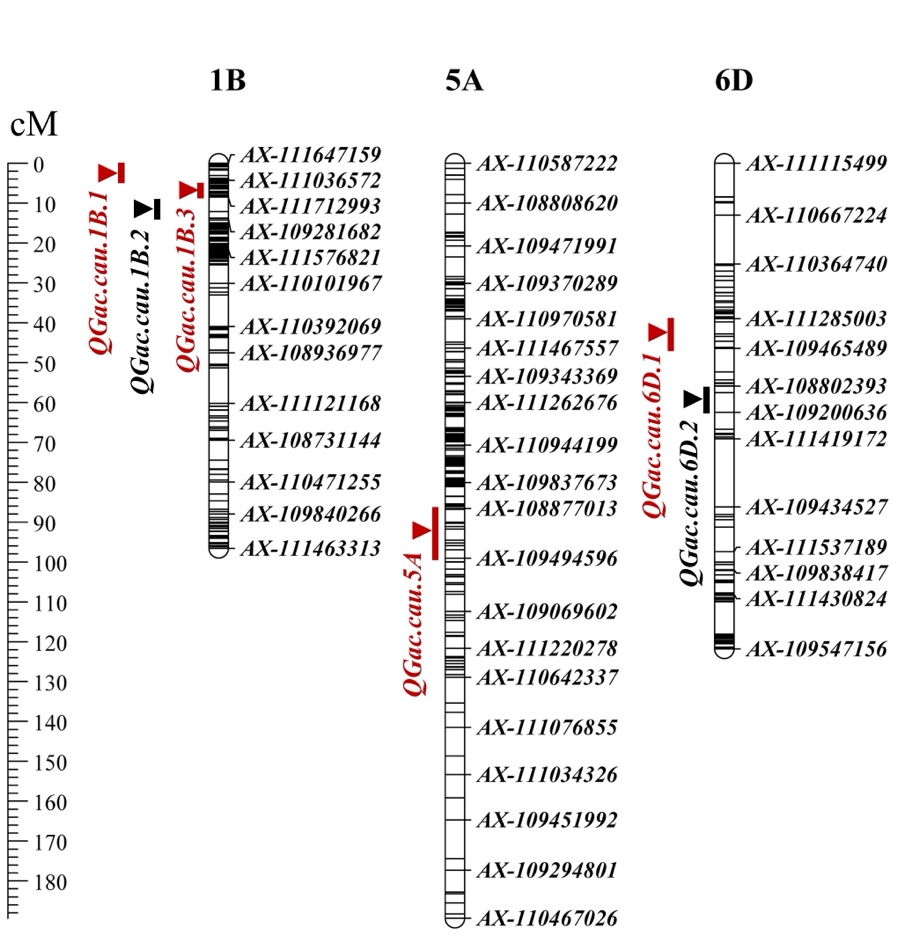


**Fig. S2** Genetic locations of QTLs associated with the grain albumin content. Uniform centimorgan (cM) scales are shown on the left, marker names are on the right. Vertical bars and triangles represent the confidence interval and the peak position for the location of each QTL, the red bars indicate environmentally stable QTLs with positive alleles from SN23，the black bars indicate environmentally stable QTLs with positive alleles from ZM17.


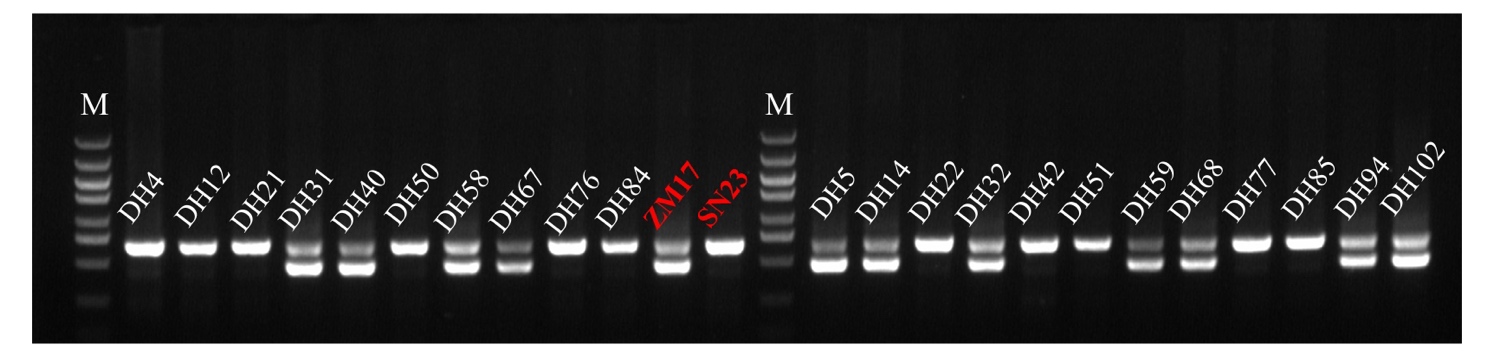


**Fig. S3** Performance of the 1BL/1RS translocation allele linked *Bam0213* marker in the two parents and partial DH lines. M, DNA marker. The sizes of the bands corresponds to SN23, it signifies 1BL/1RS translocation; conversely, if it aligns with ZM17, it indicates 1BL/1RS translocation.

# Supplementary Datasets

**Dataset S1** Primers used in this study

**Dataset S2** The mean data of GAC of DH population in four and BLUP environments

**Dataset S3** The GAC of 136 non-1BL/1RS translocation materials versus 68 1BL/1RS translocation materials

**Dataset S4** ER upregulated expression proteins relative to Pavon76

**Dataset S5** VR upregulated expression proteins relative to Pavon76
